# Editorial: Microbial regulation of soil carbon cycling in terrestrial ecosystems

**DOI:** 10.3389/fmicb.2023.1295624

**Published:** 2023-10-30

**Authors:** Mikhail Semenov, Hui Li, Yu Luo, Ye Deng, Yakov Kuzyakov

**Affiliations:** ^1^Laboratory of Soil Carbon and Microbial Ecology, Dokuchaev Soil Science Institute, Moscow, Russia; ^2^CAS Key Laboratory of Forest Ecology and Management, Institute of Applied Ecology, Chinese Academy of Sciences, Shenyang, China; ^3^Institute of Soil and Water Resources and Environmental Science, Zhejiang Provincial Key Laboratory of Agricultural Resources and Environment, Zhejiang University, Hangzhou, China; ^4^CAS Key Laboratory of Environmental Biotechnology, Research Center for Eco-Environmental Sciences, Chinese Academy of Sciences, Beijing, China; ^5^Department of Soil Science of Temperate Ecosystems, Department of Agricultural Soil Science, University of Göttingen, Göttingen, Germany; ^6^Agro-Technological Institute, Peoples Friendship University of Russia, Moscow, Russia

**Keywords:** soil microorganisms, carbon storage and sequestration, organic matter decomposition, carbon-climate feedbacks, metagenomics, ecological modeling, greenhouse gas emission

The soil organic carbon (SOC) pool is larger than the combined carbon stock in vegetation and in the atmosphere (Davidson et al., 2000; Schmidt et al., 2011; Scharlemann et al., 2014). Carbon (C) exchange between soil and the atmosphere is crucial in the terrestrial C cycle. Soil microbes regulate soil C dynamics through the transformation of plant-derived C, assimilation of C resources to build up their biomass and necromass, decomposition of soil organic matter, and CO_2_ release. The intensity and efficiency of these processes are critical determinants of net ecosystem C storage and CO_2_ flux (van Bruggen et al., 2017; Wang et al., 2021; Buckeridge et al., 2022; Camenzind et al., 2023; Chen et al., 2023; Tao et al., 2023). Although there is continuing and growing interest in elucidating the underlying microbial mechanisms driving soil C transformation, stabilization, and release processes, many challenges still remain in the manipulation of soil microbial communities for C storage. For example, these challenges include identifying the major players in C storage and decomposition; determining the genetic basis of the mechanisms involved in C sequestration; understanding complex interactions between soil physicochemical properties, plants, and microorganisms over large spatial and temporal scales; and incorporating microbial community patterns and process rates into ecosystem models (Li et al., 2021; Yang et al., 2022; Barnett and Buckley, 2023; Wu et al., 2023).

This Research Topic has aimed to increase our understanding of the role of microorganisms in soil organic C storage and mobilization processes, and also to improve our capacity to develop and evaluate cost-effective microbial strategies for C sequestration and mitigation of anthropogenic CO_2_ emissions, ultimately assisting with achievement of the goals of C neutrality and peak CO_2_. Altogether, the 11 articles published within the framework of this Research Topic cover a broad range of the processes of C cycling. Most studies have attempted to link plant communities, organic matter properties, and the soil microbiome. Liu et al. examined the characteristics of nutrient release and microbial diversity structure during the decomposition of three types of litter in arid and semi-arid regions. They revealed that the nutrient content and the rate of decomposition of mixed litter are greatly elevated compared to those of litter of single species. This is because litter mixing raises the richness and diversity of the microbial communities. Based on an original soil microcosm with decomposing corn and soy leaves, as well as soil adjacent to the leaves, Benucci et al. concluded that microbial composition was primarily affected by spatial niches, but also by soil management type and plant species in the fungal microbiome. Moisture content and pore sizes, however, were the most important drivers for bacterial communities. Guo et al. evaluated the impact of natural vegetation restoration on the physical and chemical properties of soil, as well as the diversity and composition of the microbial community, in the alkaline–saline soils of the Songnen Plain, China. *Leymus chinensis* and *Phragmites australis* grown under these harsh conditions increased SOC content and microbial diversity. Analyzing the spatial variability of soil respiration (*R*_S_) and its drivers in the Northwest Caucasus Mountains, Russia, in mixed, fir, and deciduous forests, as well as subalpine and alpine meadows, Sushko et al. concluded that microbial activity plays a crucial role in spatial variability in *R*_S_ in forests. Composition of grasses and herbs, however, was found to be the main driver of spatial variability in *R*_S_ in grasslands.

Shu et al. collected soils from degraded grasslands that had undergone 14 years of ecological restoration involving planting of shrubs with *Salix cupularis* alone or shrubs with *Salix cupularis* and mixed grasses, and compared these with soil from an extremely degraded grassland as a control. The restoration mode and its interaction with soil depth were crucial for SOC mineralization. Using metagenomics, Zhang et al. analyzed microbial carbohydrate-active enzymes in undisturbed, extensively managed, and intensively managed Moso bamboo plantations. They showed that extensive and intensive management impacts dead plant and microbial biomass decomposition and C turnover, resulting in decreased soil C. They also established that the bacterial community is the main driver of C turnover in soils under bamboo plantations.

Linkages between microbial community, C turnover processes, and potential changes in climate were examined using freeze–thaw and flood–dry cycles. Yang et al. revealed that freeze–thaw cycles in boreal forest soils activated dissolved organic matter (DOM) and increased its biodegradability, hampering C accumulation and sequestration. These findings highlight the potential of DOM molecular composition to regulate the functional states of soil bacterial communities under increased frequency and intensity of freeze–thaw cycles. Zhu et al. highlighted the fact that the shift from flooding to drying in soils collected from a riparian zone of the Three Gorges Reservoir changes keystone taxa and co-occurrence network properties, which was found to regulate soil respiration of soil aggregates.

Wang et al. studied the responses of prokaryotic, fungal, and cercozoan communities following 5 years of inundation treatments in an experimental wetland. The inundation treatments altered microbial communities in coastal wetlands, and the fungal and cercozoan communities played vital roles in regulating methane emission through microbial interactions with the methane-associated community. Jofré-Fernández et al. investigated the role of soil pH in regulating the production and consumption of reactive oxygen species (ROS) during biotic and abiotic SOM decomposition. The activities of lignin peroxidase, manganese peroxidase, and dye-decolorizing peroxidase were linked with the production of superoxide anion (${\text{O}}_{2}^{•-}$), hydrogen peroxide (H_2_O_2_), and hydroxyl radicals (•OH). The mechanisms of SOM oxidation by ROS were found to be extremely sensitive to variations in soil pH and to the stability of oxidant radicals and non-radical compounds. Li et al. investigated the occurrence of horizontal gene transfer (HGT) and established the HGT network of C metabolic genes in 764 soil-borne microbiota genomes. The inter-microbe HGT genetic traits identified in the genetic sequences of the soil-borne microbiota, as well as their involvement in the processes of metabolism and regulation of organic C, suggested the presence of pervasive and substantial effects of HGT on microbial evolution.

Overall, this Research Topic has presented evidence for the pivotal role of soil microorganisms in C cycling processes, the short- and long-term dynamics of microbial community composition, CO_2_ flux, and C storage in various ecosystems, including mixed, fir, and deciduous forests, as well as subalpine and alpine meadows, grasses, and intensively managed bamboo plantations. The contributions have expanded our understanding of the complex interplay between soil physicochemical properties, vegetation, and microbial communities at various spatial and temporal scales. These insights hold promise for the development of effective strategies for C sequestration and for the advancement of our goals of C neutrality and mitigation of anthropogenic CO_2_ emissions.

## Author contributions

MS: Writing—original draft. HL: Writing—review & editing. YL: Writing—review & editing. YD: Writing—review & editing. YK: Writing—review & editing.
